# Immediate Post-Extraction Short Implant Placement with Immediate Loading and without Extraction of an Impacted Maxillary Canine: Two Case Reports

**DOI:** 10.3390/ma14112757

**Published:** 2021-05-23

**Authors:** José Antonio Moreno-Rodríguez, Julia Guerrero-Gironés, Francisco Javier Rodríguez-Lozano, Miguel Ramón Pecci-Lloret

**Affiliations:** 1 Department of General Dentistry and Implants, Faculty of Medicine and Dentistry, University of Murcia, 30008 Murcia, Spain; joseantonio171087@gmail.com; 2 Gerodontology and Special Care in Dentistry Unit, School of Dentistry, Faculty of Medicine, University of Murcia, 30100 Murcia, Spain; fcojavier@um.es (F.J.R.-L.); miguelramon.pecci@um.es (M.R.P.-L.)

**Keywords:** short implant, impacted canine, immediate loading

## Abstract

For the treatment of impacted maxillary canines, traction associated with a complete orthodontic treatment is the first choice in young patients. However, in adults, this treatment has a worse prognosis. The surgical extraction of the impacted tooth can result in a series of complications and a compromised alveolar bone integrity, which may lead to the requirement of a bone regeneration/grafting procedure to replace the canine with a dental implant. These case reports aimed to describe an alternative treatment procedure to the surgical extraction of impacted maxillary canines in adults. Following clinical and computerized tomography-scan (CT-Scan) examination, the possibility of maintaining the impacted canine in its position and replacing the temporary canine present in its place with a dental implant was planned. A short dental implant with an immediate provisional crown was placed, without contacting the impacted canine. At 3 months follow-up, a definitive metal-ceramic restoration was placed. Follow-up visits were performed periodically. The implant site showed a physiological soft tissue color and firmness, no marginal bone loss, no infection or inflammation, and an adequate aesthetic result in all follow-up visits. These results suggest that the treatment carried out is a valid option to rehabilitate with an osseointegrated short implant area where a canine is included, as long as there is a sufficient amount of the remaining bone.

## 1. Introduction

The presence of an impacted maxillary canine is a relatively common situation, with a frequency between 1 to 3% [1,2,3]. It is more frequent in the maxillae than in the mandible in a 7:1 ratio approximately [4], and two-thirds of the impacted canines are localized in the palatal region [5].

Maintaining the impacted canine in position is associated with different complications: Peri-coronal follicular cyst, migration of adjacent teeth, tooth resorptions, pain or swelling [4,6]. Therefore, different treatment modalities have been proposed: Traction of the impacted canine associated with a complete orthodontic treatment is the treatment of choice in young patients [7]. However, in adults, the traction of the impacted canine has a worse prognosis, which worsens with age [8]. The surgical extraction of the impacted tooth compromises alveolar bone integrity, and a bone regeneration/grafting procedure may be required to replace the canine with an osseointegrated dental implant. Furthermore, surgical extraction may present a series of complications: Post-operatory swelling up to 2 days post-surgery (with a frequency of 19%), hematoma, alveolar osteitis, suppuration, postoperative pain, paresthesia, unaesthetic results, maxillary sinus communication, and adjacent tooth pulp necrosis, with a frequency from 0.5 to 2% [9,10].

Dental implants have a high long-term success rate, according to scientific evidence [11,12]. However, in patients with periodontal disease, the success of implant therapy is lower than in healthy patients, and they have a higher risk of peri-implantitis [13,14]. Although periodontitis is not a contraindication for implant treatment, its correct maintenance, and control of associated risk factors, such as tobacco, is essential in these patients [15].

The aim of these case reports was to describe an alternative treatment procedure to the surgical extraction of impacted maxillary canines in adults.

## 2. Case Reports

This study was approved by the Research Ethics Commission of the University of Murcia (ID: 2586/2019). Patients involved signed a written informed consent before carrying out the dental treatments.

### 2.1. Clinical Presentation

#### 2.1.1. Case 1

**Figure 1:**
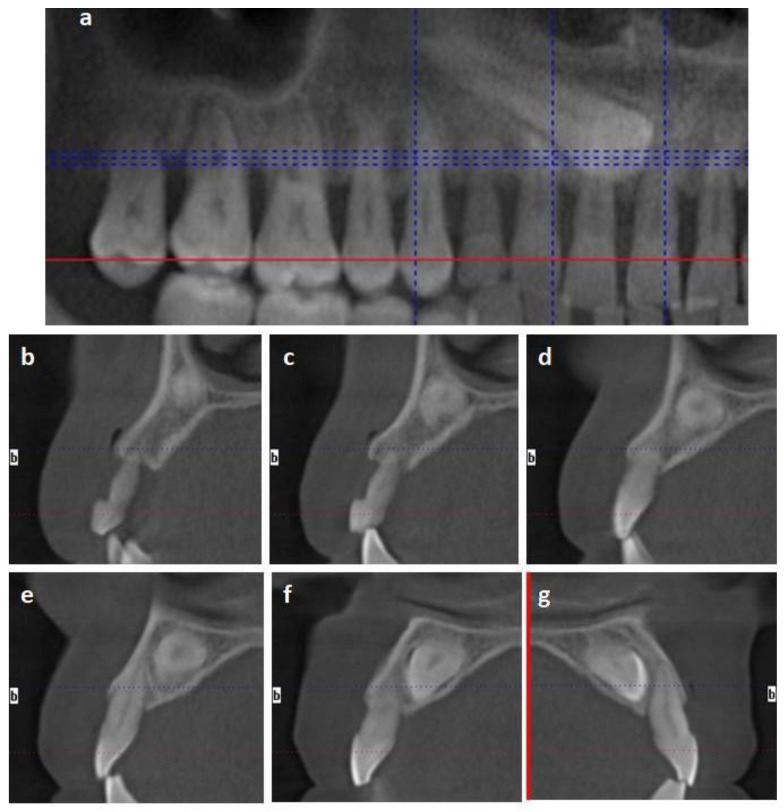
Case 1 tomography-scan (CT-Scan) images. (**a**) Image analogous to panoramic radiography of the first quadrant. (**b**–**g**) Sagittal sections show the impacted canine and its relationship with the adjacent teeth: Temporal canine 53 (**b**,**c**); tooth 12 (**d**,**e**); tooth 11 (**f**); and tooth 21 (**g**).

The patient was a healthy 50-year-old male treated in a private practice in Murcia, Spain. The patient was a smoker (20 cigarettes per day), and was diagnosed with periodontal disease Stage III (Bonelevel/CAL > 5 mm), Grade C (smoking > 10 c/d) [16], which was well-controlled (full-mouth plaque score and full-mouth bleeding ≤ 20%). The absence of tooth 13 (left maxillary canine) and the presence of the temporary canine 53 was observed in a clinical examination. The temporary tooth exhibited mobility and discomfort for the patient’s daily routine. A computerized tomography-scan (CT-Scan) examination revealed the presence of an impacted maxillary canine. The impacted tooth was located in the palatal region of the alveolar crest, and it was extended from tooth 15 to 11. The distance from the impacted canine to the other teeth varied from 0.79 to 2.55 mm. The alveolar crest configuration around the temporary and impacted canine was as follows: (1) The distance from the palatal bone peak and impacted canine was 7.72 mm; (2) The distance from the mesial and distal bone peak was 7 mm; (3) The distance from the buccal and palatal peak was 7.28 mm (Figure 1).

#### 2.1.2. Case 2

**Figure 2:**
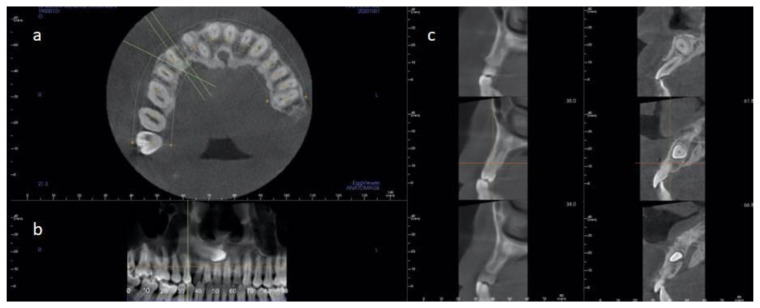
Case 2 CT-Scan images. (**a**) Axial section. (**b**) Image analogous to a panoramic radiography. (**c**) Sagittal sections show the impacted canine and its relationship with the adjacent teeth.

The patient was a healthy non-smoker 42-year-old male treated in a private practice in Elche, Spain. The patient presented a sinus tract from a temporary upper right canine that presented an advanced mobility and bleeding on probing. A CT-Scan examination revealed the presence of tooth 13 in an impacted position. The impacted canine was located in the inner region of the basal bone, and it was extended from the anterior maxillary sinus wall (above the apex of tooth 14) to tooth 21. The root and the crown were in close proximity to the premolar and central incisors’ roots. The distance from the impacted canine crown to the anterior teeth varied from 0.97 to 5.35 mm. The alveolar crest configuration around the temporary and impacted canine was (taking the lowest values) as follows: (1) The distance from the palatal bone peak and impacted canine was 9.9 mm; (2) The distance from the mesial and distal bone peak was 6.93 mm; (3) The distance from the buccal and palatal peak was 7.18 mm (Figure 2).

In both cases, the impacted canine did not exhibit any complication (i.e., root resorption, cyst, pain or swelling). The surgical extraction of the impacted canine may severely affect the alveolar crest, requiring bone regeneration/grafting techniques and damaging the vitality of adjacent teeth. Following a clinical and CT-Scan examination, the possibility of maintaining the impacted canine in position and replacing the failed temporary tooth with a dental implant was evaluated. Patients were informed on all the steps of the procedure and given informed consent before treatment initiation.

### 2.2. Clinical Procedures and Outcomes

Temporary canines were extracted, and a short dental implant was placed and provisionally-restored on the same visit. After a radiographic examination, implants were surgically placed without contact with the impacted canine at a safety distance of 2 to 3 mm. The area was locally anesthetized with an infiltrative technique and articaine 1:100,000.

After surgery, patients were prescribed 500 mg of amoxicillin every 8 h for 5 days and 600 mg ibuprofen every 8 h in the presence of pain. Furthermore, patients were instructed to rinse with chlorhexidine 0.2% twice a day for a week and avoid any trauma on the surgical area and the provisional crown during the healing period.

#### 2.2.1. Case 1

Following the extraction of the temporary canine, the socket was examined, and any granulation tissue was debrided. An implant bed was prepared using implant drills and constant irrigation with a saline solution from the apical-palatal residual socket wall. Based on the CT-Scan and the palatal bone peak as a reference, a safe 2 mm distance to the impacted canine was maintained. The temporary tooth presented a mobility grade of 2–3 and maintained a minimal portion of its root structure within the alveolar bone (<2 mm). Therefore, the width of the alveolar bone up to the included canine was kept intact for drilling and implant stabilization. All the surgical procedures were performed through the socket without elevating any surgical flap. The dental implant (IPX, Galimplant, Lugo, Spain) (4 × 6 mm size) platform was placed at the mesial and distal bone peak (slightly supra-osseous to the distal bone peak and slightly infra-osseous to the mesial bone peak), despite being an implant with its entire surface treated for its intraosseous placement. However, the soft tissue width was 5 mm. Scientific evidence shows that an adequate soft tissue width helps preserve the crestal bone [17,18,19]. Submerging the implant when the definitive abutment is not used leads to marginal bone loss for stabilizing the peri-implant biological width due to the repeated insertion and reinsertion of the provisional during the prosthetic phase [20].

**Figure 3:**
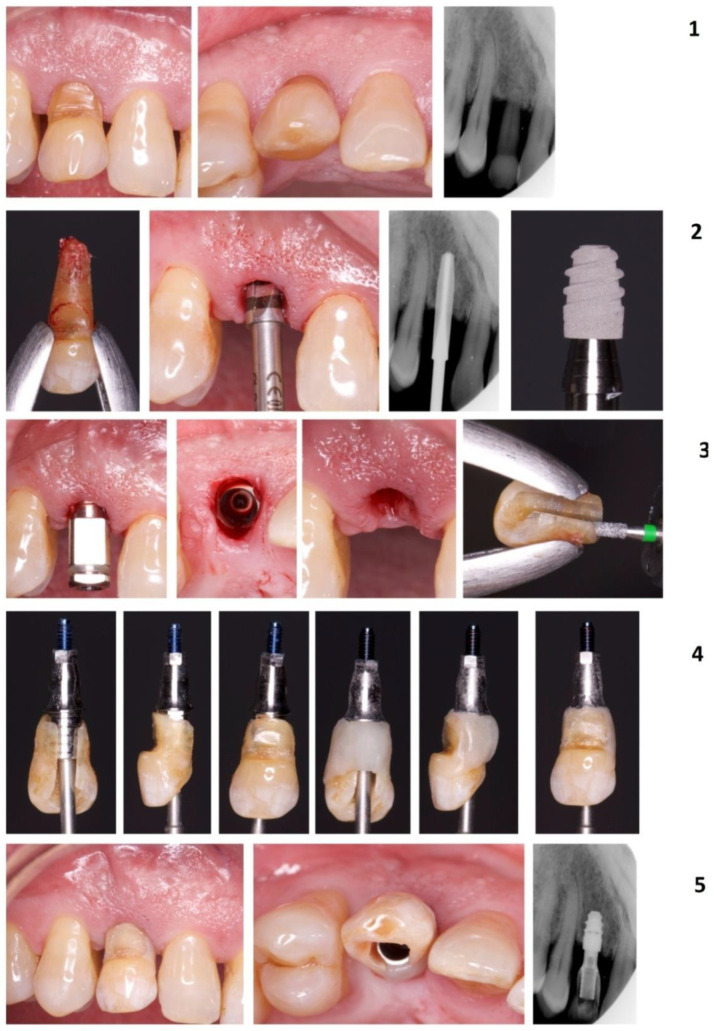
The complete sequence of extraction of the temporal canine and provisionalization phase (Case 1). Temporary canine (images in row 1). Implant placement and implant (Galimplant IPX, size 4 × 6 mm) (images in row 2). The temporary canine crown was adapted and used as a provisional restoration connected to the implant with a titanium screw provisional abutment (rows 3 and 4). Provisional placement and periapical radiograph (row 5).

As a consequence of periodontal disease evolution, the patient presented a wider band of supra-alveolar soft tissue. No gaps were present, and no soft or bone tissue augmentation techniques were required. The temporary canine crown was adapted and used as a provisional restoration, which was connected to the implant with a titanium screw provisional abutment. The provisional crown sealed the socket, and no sutures were required. The provisional prosthetic restoration of the implant was carried out immediately after the implant was placed, but it lacked load, being freed of all types of occlusal forces in maximum intercuspation and lateral movements (Figure 3).

**Figure 4:**
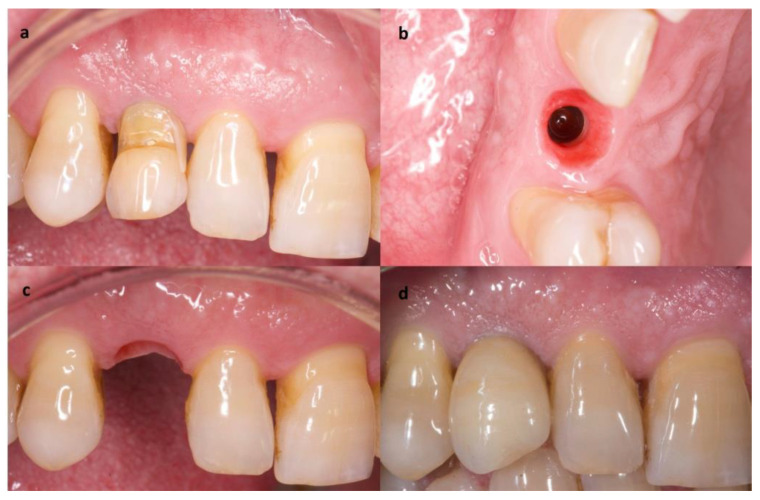
Definitive crown placement process (Case 1). (**a**) Provisional crown made with the temporary canine. (**b**) Occlusal view of adequate soft tissue. (**c**) Buccal view of adequate soft tissue. (**d**) Definitive crown.

**Figure 5:**
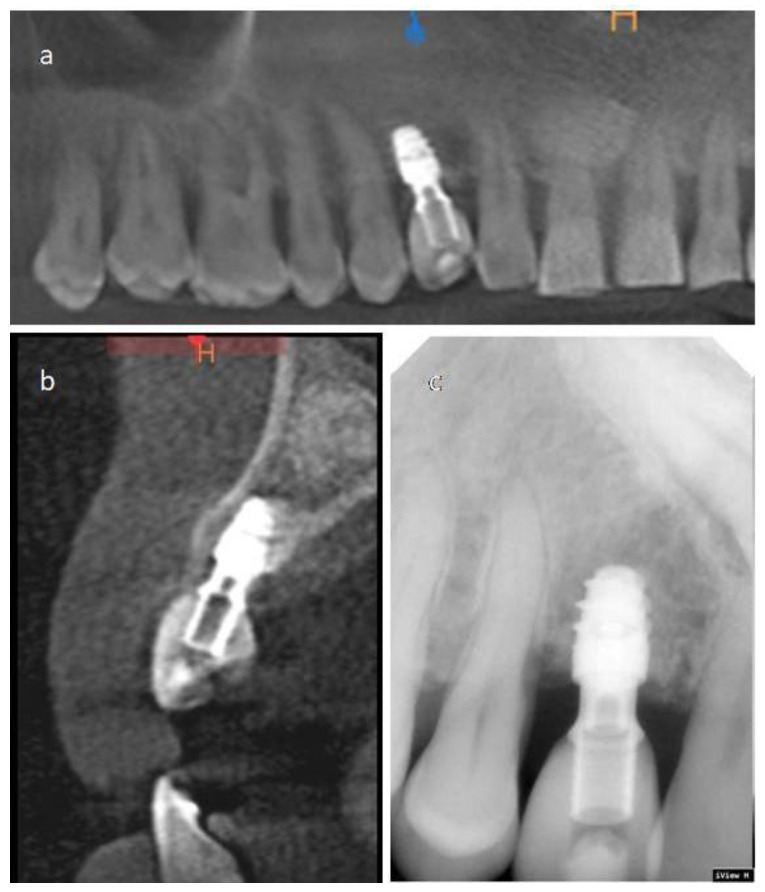
Case 1 last follow-up (3 years after the surgery). (**a**) CT-Scan image analogous to panoramic radiography of the first quadrant. (**b**) Sagittal section shows the dental implant. (**c**) Periapical radiograph.

After a week, the area showed optimal wound healing, and the patient reported no pain or swelling. At 3 months follow-up, a definitive metal-ceramic restoration was placed (Figure 4). Follow-up visits were scheduled every 3 months along the first year and every 6 months after the first year. The implant site showed a physiological soft tissue color and firmness, no marginal bone loss, no infection or inflammation, and an adequate aesthetic result in all follow-up visits. The last follow-up visit was scheduled 3 years after the surgery, and no changes were observed from the day of the placement of the definitive crown (Figure 5).

#### 2.2.2. Case 2

**Figure 6:**
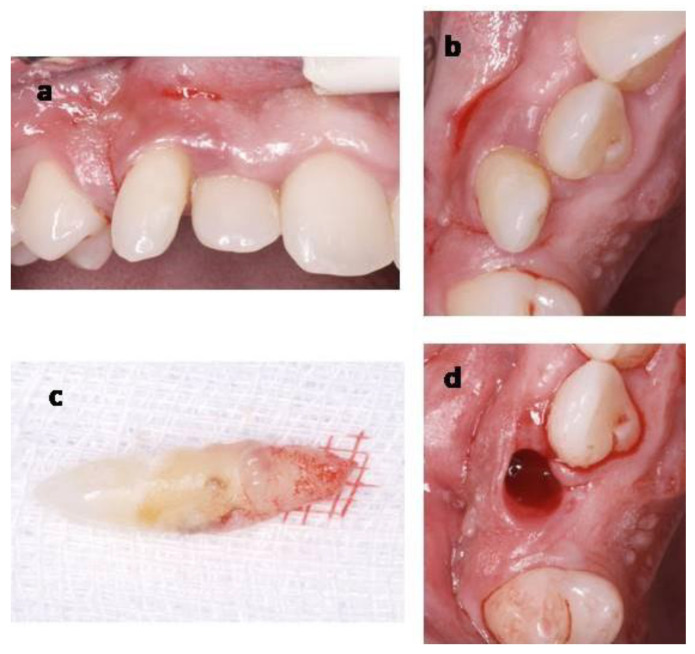
Temporary canine extraction (Case 2). (**a**) Buccal photography of temporary canine. (**b**) Occlusal photography of the temporary canine. (**c**) Extracted temporary canine. (**d**) Extraction socket.

**Figure 7:**
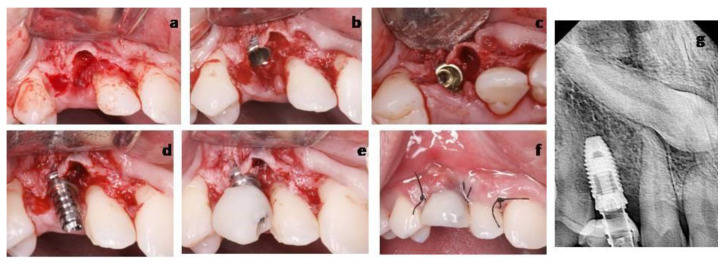
Implant and provisional crown placement (Case 2). (**a**) Abuccal full-thickness flap was raised until the buccal alveolar crest was exposed. (**b**) Inserted implant: Apically from the cementoenamel junction of adjacent teeth. (**c**) Implant occlusal view. (**d**) Implant abutment. (**e**) Provisional screw resin crown. (**f**) Flap was adapted and sutured. (**g**) Periapical radiograph.

This case followed the aforementioned procedure, with a series of differences. A temporary canine was extracted (Figure 6). A sulcular incision was performed around the temporary canine and extended to the adjacent teeth, both mesially and distally. A buccal full-thickness flap was raised until the buccal alveolar crest was exposed, in order to increase the visibility and access, since the planned implant position and the temporary canine socket did not coincide. The implant bed preparation was done distally from the residual socket, and an implant (NobelParallel, Nobel Biocare, Gothenburg, Sweden) (4.3 × 7 mm in size) was inserted approximately 4 mm apically from the cementoenamel junction of adjacent teeth. No soft tissue or bone graft was required. In this case, a dehiscence <2 mm remains since is not treated by regenerative bone techniques. In the same way as in Case 1, an adequate band of keratinized and inserted tissue (>2 mm) and soft tissue thickness (>2 mm) guarantees the stability of the bone crest over time [17,18,19]. A provisional screw resin crown was designed. Finally, the flap was adapted and sutured (Figure 7). The follow-up visit scheme was the same as those described in Case 1. The last follow-up visit was scheduled 2 years post-surgery, and no inflammation, pain, bleeding on probing or any changes were shown from the visit in which the definitive restoration was placed.

## 3. Discussion

These case reports present two procedures of immediate post-extraction short implant placement with immediate loading, without the extraction of impacted maxillary canines. An impacted canine orthodontic traction or surgical extraction in failed orthodontic cases is the first treatment choice, due to the risk of maintaining the impacted canine, mainly when a peri-coronal cyst or root resorptions are present [21,22,23]. However, in adults, the presence of asymptomatic ankylotic impacted canines with years of evolution is a common clinical situation [24]. Furthermore, when the impacted canine occupies the central area of the basal bone, and is related to the maxillary sinus cavity or in the proximity of the apex of adjacent teeth, an alternative treatment without the extraction of the impacted tooth should be considered. For example, when the adjacent area is available for implant placement, such as edentulous maxillae with impacted canines, a computerized three-dimensional planning system can be used to place implants that avoid contact with the impacted canine [25]. In addition, a series of cases among the literature in which implants were placed through the impacted teeth led to an adequate cicatrization [26].

Short implants in implantology are a suitable therapeutic alternative to support single fixed restorations. The implants’ shorter length presents several advantages for both the practitioner and patient, such as less complex surgeries, minor discomfort, morbidity, treatment time, and cost [27,28]. Current studies among the literature report a high success rate and fewer complications associated to the placement of short implants than standard-length dental implants with bone augmentation/grafting [29,30]. On the other hand, other studies suggest that implants with less than 8 mm in length have a higher risk of failure, added to the influence of various factors associated with peri-implant tissue and prosthetic rehabilitation [28,31]. Short implants are used mainly to avoid sinus lift elevations and inferior alveolar nerve damage or avoid complex surgeries in medically compromised patients [27]. One of its uses may be to avoid contact with the included teeth.

Maintaining an impacted canine in its place and restoring the function with a short osseointegrated implant without contact with it has been proposed and described in a previous case report [32]. However, no provisional crown was connected until the end of the osseointegration period. In these reports, short implants were provisionalized immediately. Immediate implant placement and immediate anterior loading are recommended procedures, which are supported by an extensive scientific literature in modern dentistry [33,34]. Furthermore, the placement of an immediate flapless implant significantly reduces patient discomfort. Lastly, immediate loading has also shown to influence the final aesthetic results by providing support to soft tissue and avoiding marginal soft tissue collapse and loss [33,35,36].

Case 1 shows a patient with periodontitis and also a 20 c/d smoker. Despite not eliminating the risk factor for the stability of peri-implant health that tobacco supposes [37], this is a patient with periodontitis controlled by constant visits for patient supervision, reinforcement of oral hygiene, and non-surgical maintenance treatment. Therefore, periodontitis, regardless of stage and grade, does not seem to be a contraindication to carry out the proposed treatment protocol, as long as there is proper maintenance and control of the periodontal disease [15].

## 4. Conclusions

Within the limitations of the present study, the reported cases present a maintenance of impacted maxillary canines without root resorptions, cysts or any other complications related to the impacted teeth. The aesthetic and function may be restored with short osseointegrated implants without contact with the impacted canine and an immediate provisionalization. Soft and bone tissues were preserved, and no grafting procedures were required. Furthermore, in a follow-up period between 2 to 3 years, no soft tissue alterations, marginal bone loss, bleeding or non-physiological probing depths or infections were observed.

## Data Availability

Data is contained within the article.
